# Chronic limb sensory abnormalities caused by retained intraspinal metallic foreign body: a case report

**DOI:** 10.3389/fmed.2026.1886952

**Published:** 2026-08-10

**Authors:** Qi Xiao, Jianjun Li, Licheng Huang, Yang Lin, Lei Zhu, Xiangxin Zhong, Tao Wang, Zhifeng Wang, Baibai Ye, Wenguang Fang

**Affiliations:** 1 The Sixth People's Hospital of Huizhou, Huizhou, Guangdong, China; 2 Hunan University of Chinese Medicine, Changsha, Hunan, China

**Keywords:** acupuncture, intraspinal foreign body, metallic foreign body, spinal cord compression, surgical removal

## Abstract

Intraspinal foreign body retention is a rare clinical entity, usually resulting from trauma, surgery, or invasive procedures, and may lead to progressive neurological dysfunction. We report a patient who presented with burning pain in the right sole and bilateral lower limb weakness, and was diagnosed with a retained metallic foreign body in the thoracic spinal canal by imaging examinations. We discuss the clinical features, imaging diagnosis, and surgical management based on the diagnostic and therapeutic process of this case. This report indicates that invasive therapies such as acupuncture carry the potential risk of needle breakage, migration, and subsequent intraspinal foreign body retention, which deserves clinical attention. Strict standard operation during acupuncture is crucial to avoid such iatrogenic complications.

## 1 Introduction

Intraspinal foreign body retention is an uncommon clinical entity that poses considerable diagnostic and therapeutic challenges (1). Metallic foreign bodies represent the most prevalent type encountered in clinical practice. Generally, clinical findings associated with intraspinal foreign bodies vary widely, ranging from localized axial back pain and radiculopathy to progressive myelopathy, depending on the anatomical location and the degree of neural compression. When left within the spinal canal for an extended period, such foreign bodies act as a persistent space-occupying lesion that compresses neural elements. They also trigger local inflammatory reactions, fibrous hyperplasia, capsule formation, and may even lead to secondary infection or granulomatous lesions. Consequently, patients often experience gradual neurological decline, presenting with sensory deficits, motor weakness, and sphincter dysfunction (2, 3).

Acupuncture, as a core component of Traditional Chinese Medicine (TCM), is deeply integrated into the national healthcare system and widely utilized for clinical management in China (4–6). Although iatrogenic intraspinal foreign body retention related to acupuncture is rare, this serious complication remains underdiagnosed and deserves increased clinical attention (7, 8). Unlike foreign bodies introduced by high-energy acute trauma or open surgery, the presence of a broken acupuncture needle in the dural sac is uniquely insidious and can result from two primary mechanisms. While thin broken needles can theoretically migrate silently through fascial and muscular layers over time, direct breakage within the spinal canal—often secondary to excessively deep insertion combined with sudden patient movement during therapy—is considered a highly probable mechanism. Regardless of the precise mechanism, these retained metallic fragments cause chronic micro-irritation, leading to delayed presentation and non-specific symptoms such as chronic paresthesia and progressive paresis of the extremities, which creates a substantial diagnostic pitfall. Specific clinical identification heavily relies on advanced imaging, with computed tomography (CT) being highly sensitive to metallic fragments, while magnetic resonance imaging (MRI) is essential for evaluating spinal cord damage and surrounding tissue reactions, despite the potential interference of metallic susceptibility artifacts.

The aim of this study is to report a rare case of a broken acupuncture needle in the thoracic spinal canal. By highlighting its unique clinical and radiological characteristics, we intend to raise awareness of this delayed and iatrogenic complication, and provide insights into its diagnosis and surgical management.

## 2 Case presentation

A 51-year-old female was admitted to our hospital on January 20, 2025. Her clinical timeline began more than 1 year prior to admission when she initially developed numbness in both hands, pre-dominantly at the fingertips, alongside a burning sensation in her soles. Initial conservative treatments with oral neurotrophic agents, such as mecobalamin and B vitamins, yielded poor results. Consequently, she received long-term routine physical therapy such as massage, moxibustion, and traditional acupuncture, which provided only transient and partial relief.

Notably, 2 months prior to admission, driven by intractable and worsening burning pain in her soles, the patient underwent a high-risk acupuncture intervention that exceeded standard clinical practice. She explicitly declined to disclose the specific procedural details of this session. Following this intervention, she experienced no symptomatic relief. Instead, she progressively developed entirely new neurological deficits. These included new-onset weakness in both lower limbs and a neuropathic burning pain that ascended from her soles to the proximal regions of her lower extremities. These deficits were accompanied by significant difficulty in squatting, standing up, and climbing stairs. She denied experiencing sudden falls, gait instability, a cotton-wool sensation, or sphincter dysfunction. Given this progressive neurological decline, she was admitted to the Department of Neurology for further evaluation.

## 3 Diagnostic assessment

On physical examination, multiple punctate skin scars were observed over the spinous processes from the cervical to lumbar spine. Light touch and pinprick sensations were decreased at the fingertips of both hands. Motor examination of the lower extremities revealed that muscle strength for left hip flexion and left knee extension was graded as IV, and the right extensor hallucis longus was also graded as IV. Muscle strength in the remaining lower limb muscle groups was normal. Sensory examination demonstrated decreased vibratory sensation in the right lower extremity and hyperesthesia to touch and pain on the right sole. Deep tendon reflexes of both lower limbs were symmetric and normoactive, and pathological reflexes were absent. Furthermore, there was no saddle anesthesia, and the patient had intact sphincter function with no bowel or bladder dysfunction.

Given the multisegmental, asymmetric, and atypical nature of the patient's neurological symptoms, establishing a definitive diagnosis upon admission was clinically challenging. Prior to completing spinal imaging (MRI and CT), the neurologists prioritized a comprehensive laboratory workup to systematically rule out severe systemic etiologies. First, to exclude occult malignancies that could cause spinal nerve dysfunction via metastasis, direct invasion, or paraneoplastic syndromes, a comprehensive tumor marker panel (including AFP, CEA, FER, CA19-9, CA15-3, CA72-4, and CA125) was performed to screen for hepatic, gastrointestinal, breast, and reproductive system tumors, which are prevalent in females. Second, rheumatology and autoimmune panels (including an ANA 7-item panel, rheumatoid factor, and HS-CRP) were conducted to rule out autoimmune-mediated neuropathies or connective tissue diseases that can present with complex sensorimotor deficits. Finally, routine evaluations—including complete blood count, liver and renal function, electrolytes, fasting blood glucose, coagulation, and thyroid function—were performed to assess the patient's baseline physiological status and prepare for potential surgical intervention. The laboratory results for tumor markers, liver and renal function, electrolytes, blood glucose, thyroid function, coagulation, and autoimmune antibodies were all within normal reference ranges, effectively ruling out tumor-induced or autoimmune neural compromise. However, notable findings included a slightly elevated erythrocyte sedimentation rate and mild microcytic hypochromic anemia. The detailed laboratory parameters and results are summarized in Table 1, and the complete raw laboratory reports are provided in Supplementary Tables S1–10.

**Table 1:** Summary of routine laboratory examinations upon admission.

Examination category	Specific parameters	Results/values
Complete blood count	WBC, RBC, HBG, PLT, NEU%, LYM%, etc.	Mild anemia; thrombocytosis
Liver function	ALT, AST, ALP, GGT, LDH, TBA	Normal
Renal function	BUN, CREA, UA	Normal
Electrolytes	K^+^, Na^+^, Cl^−^	Normal
Blood glucose	GLU	Normal
Tumor markers	AFP, CEA, FER, CA-125, CA-15-3, CA-19-9, CA72-4	Normal
Thyroid function	FT3, FT4, TSH	Normal
Autoimmune/rheumatology	ANA 7-item panel, HS-CRP, RF, ASO, CCP	Normal
Coagulation function	PT, INR, APTT, Fib, TT	Normal
Inflammatory marker	ESR	Normal

**Figure 1:**
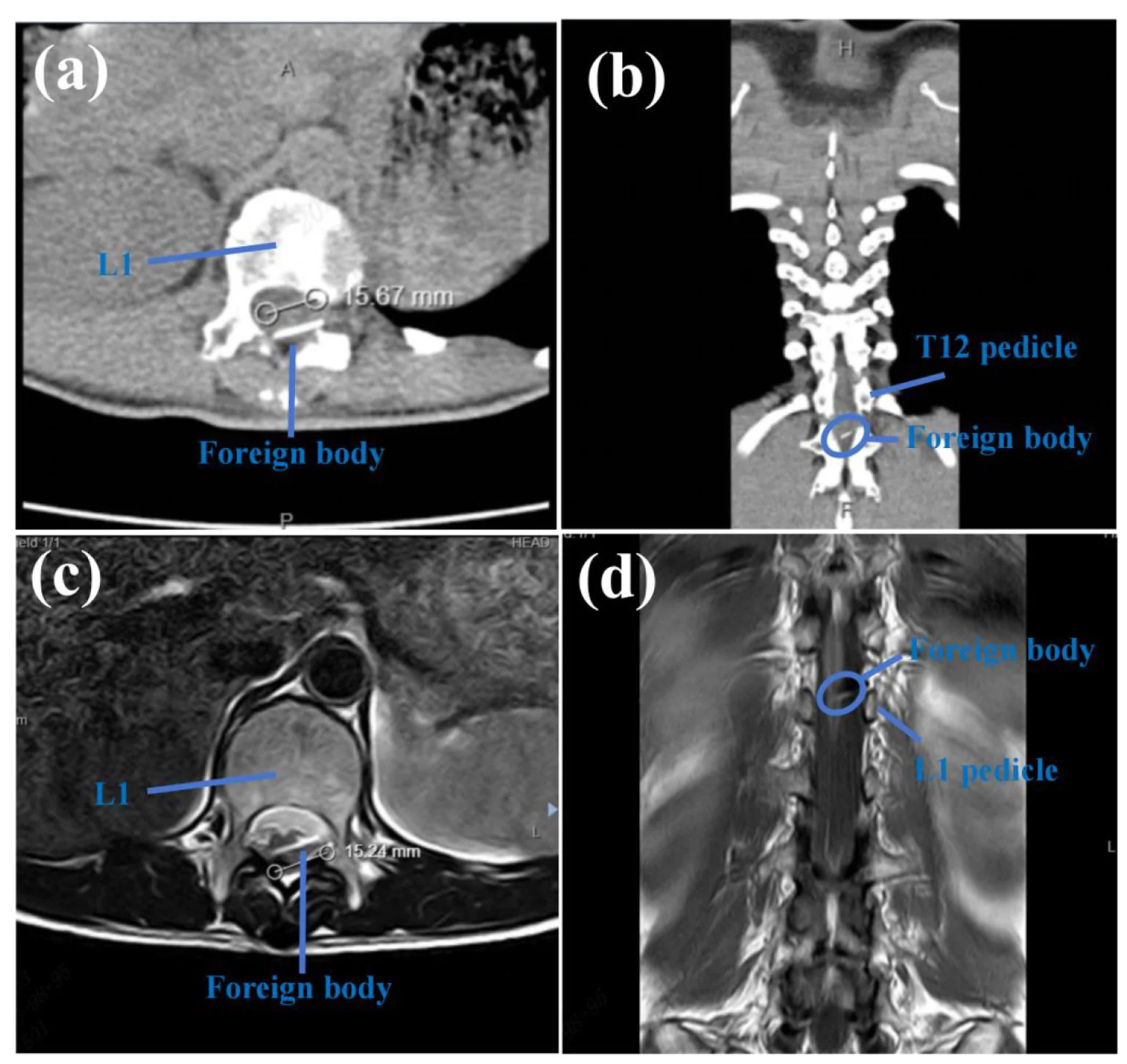
Pre-operative spinal imaging demonstrating the retained metallic foreign body. **(a)** Axial and **(b)** coronal CT images showing a high-density linear metallic foreign body at the T12-L1 level, measuring 15.67 mm in length. **(c)** Axial and **(d)** coronal T2-weighted MRI confirming the precise intradural location of the lesion, measuring 15.24 mm in length. Vertebral levels are explicitly labeled to facilitate anatomical localization.

Spinal CT and MRI were performed, demonstrating a linear metallic foreign body within the spinal canal at the T12-L1 level (Figure 1). Specifically, coronal and axial CT images clearly revealed a high-density linear object measuring 15.67 mm in length. Corresponding coronal and axial T2-weighted MRI further confirmed the exact location of the lesion, measuring 15.24 mm with well-defined margins. The slight discrepancy in the measured lengths between the two modalities is primarily attributed to magnetic susceptibility artifacts inherent to MRI when imaging metallic objects. Consequently, the CT-derived measurement (15.67 mm) is considered the more accurate representation of the foreign body's true dimensions. Based on these imaging findings, a preliminary diagnosis of an intraspinal retained metallic foreign body at T12-L1 was made.

**Figure 2:**
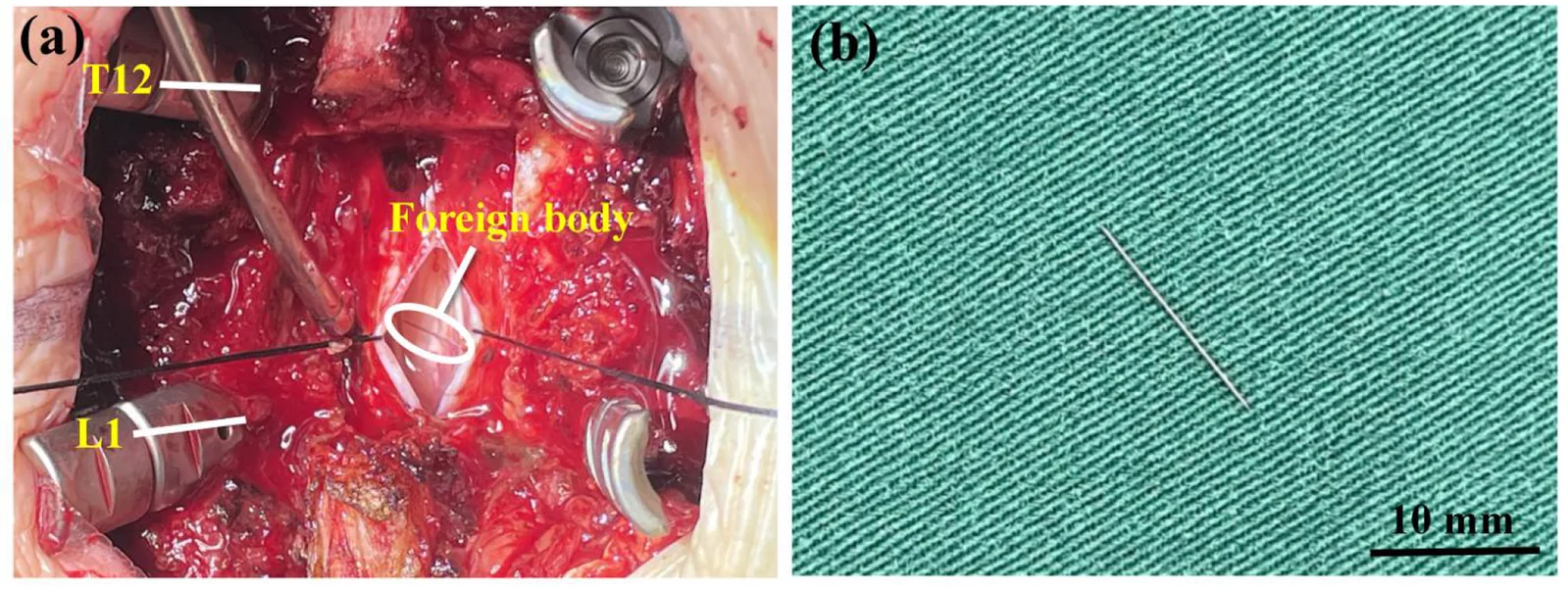
**(a)** Intraoperative view of the opened spinal canal; **(b)** Close-up of the removed foreign body, Scale: 10 mm.

After initial assessment in the Department of Neurology, the patient was transferred to the Department of Spinal Surgery. On January 24, 2025, the patient underwent a single-level laminectomy for intradural foreign body extraction, combined with a short-segment instrumented spinal fusion (Figure 2).

Briefly, via an 8-cm posterior midline incision, the posterior elements of T12 and L1 were exposed. To safely access the spinal canal, a single-level laminectomy involving the resection of the T12 spinous process, lamina, and partial facet joints was performed. Upon dural opening, a needle-like metallic foreign body was identified and successfully extracted. Notably, no obvious local tissue proliferation or adhesions were observed around the foreign body intraoperatively, and therefore, no tissue was submitted for histopathological examination. Primary dural closure was performed with 5-0 absorbable sutures, and no cerebrospinal fluid leakage was detected.

To prevent iatrogenic spinal instability and subsequent degeneration resulting from the resection of the T12 posterior stabilizing structures, a short-segment (T12-L1) interbody pedicle screw-rod fixation system was implanted under C-arm fluoroscopic guidance. Postoperative CT confirmed the complete extraction of the foreign body and the optimal positioning of the internal fixation. To accurately represent the dimensions of the foreign body, precise measurements were digitally marked on the postoperative CT imaging.

Then, the patient received neurotrophic agents, B vitamins, and non-steroidal anti-inflammatory drugs for analgesia for 3 weeks. During this period, clinical symptoms showed no significant improvement compared with the pre-operative state, and difficulty in squatting persisted. At the fourth week, a comprehensive rehabilitation protocol was initiated, including syndrome-differentiated acupuncture, local red-light irradiation, and moxibustion. By the fifth week, subjective reports of numbness in both hands and burning pain in the right sole had markedly decreased, and squatting difficulty was also alleviated. The patient's condition remained stable, and she was subsequently discharged.

## 4 Discussion

### 4.1 Analysis of the pathophysiological mechanism of limb symptoms

In this case, the patient presented with progressively worsening burning pain extending from the proximal right lower limb to the sole, accompanied by bilateral lower limb weakness and difficulty with squatting and standing. These manifestations were multisegmental, asymmetric, and showed gradual aggravation over time. The metallic foreign body, measuring approximately 4 cm in length, was located at the T12/L1 level, corresponding anatomically to the conus medullaris and lumbosacral nerve roots (9). Clinically, the patient exhibited asymmetric sensory and motor deficits without saddle anesthesia, sphincter dysfunction, or abnormal deep tendon and pathological reflexes. This specific clinical pattern indicates that the patient did not develop a complete conus medullaris syndrome. Instead, these findings perfectly correspond to an early, localized, and asymmetric irritation of the descending lumbosacral nerve roots within the dural sac.

In contrast to acute penetrating injuries reported in previous literature, long-term retention of a foreign body within the spinal canal can induce chronic aseptic inflammation, disturb the local neural microenvironment, and activate nociceptive pathways, thereby leading to intractable neuropathic pain. The patient's severe and refractory burning sensation in the right sole, which showed minimal response to conventional analgesic treatment, is consistent with this pathophysiological pattern.

### 4.2 Mechanism of intradural retained foreign body

The presence of a broken acupuncture needle within the intradural space is an exceptional complication. Based on the patient's clinical timeline and consultations with experienced acupuncture practitioners, two potential biomechanical mechanisms could explain this phenomenon.

The first, and highly probable scenario in this case, involves direct iatrogenic breakage within the spinal canal. During the deep and intensive acupuncture session 2 months prior to admission, the practitioner may have advanced the needle excessively deep, penetrating the interlaminar space and entering the spinal canal. Any sudden spinal movement or postural shift by the patient during needle retention could exert immense shearing forces on the ultra-thin needle, causing it to snap directly within the dural sac. This mechanism perfectly aligns with the patient's sudden onset of entirely new, progressive lower limb symptoms immediately following that specific session.

A second potential mechanism is delayed, gradual intradural migration. If the needle initially broke and lodged in the relatively superficial paraspinal tissues, its solid, sharp-tipped profile would facilitate deep tissue tracking. The relatively restricted mobility of the thoracic spine may have prevented macroscopic bending of the needle, thereby avoiding severe early-stage mechanical back pain. Consequently, the patient did not seek immediate medical attention. Driven by the unidirectional forces of continuous paraspinal muscle contractions during daily activities, the rigid needle could slowly migrate deeper along tissue planes, eventually penetrating the ligamentum flavum and dura mater, until it caused significant radicular symptoms.

We reviewed the literature to compare this exceptional event with other reported intraspinal foreign bodies. Similar cases are exceedingly rare and pre-dominantly involve retained broken surgical instruments (8) or ballistic fragments from gunshot wounds (10). Unlike ballistic fragments, which enter with high kinetic energy and typically cause immediate, massive traumatic cord injury, or surgical instruments broken during open visualization, a retained acupuncture needle is uniquely insidious. Its microscopic diameter and sharp profile allow for either deep initial penetration without immediate massive hemorrhage or gradual silent migration, resulting in chronic micro-irritation and delayed neuropathic pain.

### 4.3 Analysis of the mechanism of neurological recovery and the role of rehabilitation

The patient showed only limited neurological improvement in the early postoperative period, with clinical symptoms remaining static during the initial 3 weeks of standard pharmacological management. This delayed recovery may be explained by several complementary mechanisms: first, prolonged neural compression can lead to reversible neural dysfunction that does not resolve immediately after decompression (11, 12); second, postoperative local edema and inflammatory reactions may temporarily counteract the beneficial effects of surgical decompression (13); third, activation of central sensitization may independently maintain pain perception despite removal of the offending lesion (14).

Subsequently, a more pronounced neurological improvement was observed starting from the fourth week, which coincided with the initiation of a combined traditional Chinese medicine rehabilitation protocol, including acupuncture, moxibustion, and red-light irradiation. However, nerve recovery following decompression is a complex physiological process that occurs naturally over time. While the rehabilitation protocol was intended to promote local blood circulation and neural metabolic recovery, it is difficult to definitively differentiate its specific therapeutic contribution from the natural postoperative recovery trajectory in this single-case setting. Thus, while the concurrent initiation of rehabilitation and symptom resolution suggests a potential synergistic effect, this observation requires cautious interpretation, and further controlled studies are needed to clarify its exact role.

### 4.4 Limitations

Several limitations of this study must be acknowledged. First, as a single case report, the generalizability of the present observations is inherently limited. Second, due to institutional equipment constraints, electrophysiological evaluations were not performed. The absence of these tests precluded precise pre-operative localization of the involved nerve segments and limited objective evaluation of postoperative functional recovery. Consequently, the proposed pathophysiological mechanisms are derived primarily from clinical and radiological findings and remain deductive rather than experimentally verified. Similarly, although the patient exhibited clinical improvement following postoperative rehabilitation therapy, this single experience cannot rule out the natural recovery process and does not support broad clinical recommendations or standardized treatment protocols. Definitive diagnostic and therapeutic strategies for intraspinal retained metallic foreign bodies will require larger-scale, multi-center observational studies.

## 5 Conclusion

Intraspinal intradural metallic foreign body retention is an extremely rare condition that is easily misdiagnosed due to its non-specific and insidious manifestations. Early surgical removal is recommended once the diagnosis is confirmed, and postoperative rehabilitation is essential for optimal neurological recovery. In patients with unexplained chronic spinal cord symptoms and a history of acupuncture, intraspinal foreign body should be included in the differential diagnosis.

This case demonstrates that broken acupuncture needles can migrate into the spinal canal and present as delayed, chronic neurological deficits caused by long-term local irritation. Although surgical extraction relieves mechanical compression, neurological recovery may be prolonged. As a single case report, this study cannot establish general guidelines; however, it enhances clinical awareness and provides a valuable reference for delayed iatrogenic intraspinal foreign bodies.

## Data Availability

The original contributions presented in the study are included in the article/supplementary material, further inquiries can be directed to the corresponding authors.
