# Supplementary material for: Chronic limb sensory abnormalities caused by retained intraspinal metallic foreign body: a case report
Source: Front Med (Lausanne). 2026 Aug 10;13:1886952. doi: 10.3389/fmed.2026.1886952 (PMC13500284; doi:10.3389/fmed.2026.1886952)
Supplement: Supplementary file 1 [file Supplementary_file_1.docx]

Table S1. The results of complete blood count with 5-part differential

| **Inspection items** | **Detection results** | **Units** | **Reference ranges** |
| --- | --- | --- | --- |
| WBC | 5.19 | 10^9/L | 3.5-9.5 |
| NEU# | 3.67 | 10^9/L | 1.8-6.3 |
| LY# | 0.90 | 10^9/L | 1.1-3.2 |
| MO# | 0.46 | 10^9/L | 0.1-0.6 |
| EOS# | 0.12 | 10^9/L | 0.02-0.52 |
| BAS# | 0.04 | 10^9/L | 0.00-0.06 |
| NEU% | 70.6 | % | 40-75 |
| LYM% | 17.4 | % | 20-50 |
| MO% | 8.8 | % | 3-10 |
| EOS% | 2.4 | % | 0.4-8 |
| BAS% | 0.8 | % | 0-1 |
| RBC | 3.80 | 10^12/L | 3.8-5.1 |
| HBG | 98 | g/L | 115-150 |
| HCT | 29.9 | % | 35-45 |
| MCV | 78.6 | fL | 82.0-100 |
| MCH | 25.7 | Pg | 27-34 |
| MCHC | 328 | g/L | 346-354 |
| PLT | 402 | 10^9/L | 125-350 |
| MPV | 8.7 | fL | 7.4-12.5 |
| PDW | 15.8 |  | 10.5-18.1 |
| PCT | 0.351 |  | 0.108-0.282 |
| RDW-SD | 16.7 |  | 11-16 |
| RDW-CV | 48.9 |  | 35-56 |

Table S2. The results of erythrocyte sedimentation rate (ESR)

| **Inspection Items** | **Detection results** | **Units** | **Reference ranges** | **Test Method** |
| --- | --- | --- | --- | --- |
| ESR | 52 | mm/h | 0-20 | Manual Method |

Table S3. The results of liver enzyme panel (6 tests)+fasting blood glucose (FBG)

| **Inspection items** | **Detection results** | **Units** | **Reference ranges** | **Test Method** |
| --- | --- | --- | --- | --- |
| ALT | 31.4 | U/L | 13-35 | Malate Dehydrogenase Method |
| AST | 25.0 | U/L | 7-40 | Lactate Dehydrogenase Method |
| ALP | 73.90 | U/L | 35-135 | Continuous Monitoring Method |
| GGT | 17.60 | U/L | 7-45 | Enzymatic Method |
| LDH | 164.6 | U/L | 120-250 | Lactate Substrate Method |
| TBA | 6.48 | umol/L | 0-21 | Diazonium Salt Method |
| GLU | 4.57 | mmol/L | 3.9-6.2 | Hexokinase Method |

Table S4. The results of renal panel (3 assays) +serum electrolyte panel (3 tests)

| **Inspection items** | **Detection results** | **Units** | **Reference ranges** | **Test Method** |
| --- | --- | --- | --- | --- |
| BUN | 4.08 | mmol/L | 2.5-6.4 | Urease Method |
| CREA | 56.1 | umol/L | 44-80 | Enzymatic Method |
| UA | 311.6 | umol/L | 154.7-357.0 | Uricase Method |
| K⁺ | 4.66 | mmol/L | 3.5-5.3 | Ion-Selective Electrode Method |
| Na⁺ | 136.9 | mmol/L | 135-147 | Ion-Selective Electrode Method |
| Cl⁻ | 100.6 | mmol/L | 99-110 | Ion-Selective Electrode Method |

Table S5. The results of free T3 / free T4 / TSH Panel

| **Inspection items** | **Detection results** | **Units** | **Reference ranges** | **Test Method** |
| --- | --- | --- | --- | --- |
| FT3 | 3.99 | pmol/L | 2.76-6.45 | Chemiluminescence Method |
| FT4 | 16.90 | pmol/L | 11.2-23.81 | Chemiluminescence Method |
| TSH | 1.156 | MIU/L | 0.35-5.10 | Chemiluminescence Method |

Table S6. The results of coagulation function panel (5 assays)

| **Inspection items** | **Detection results** | **Units** | **Reference ranges** | **Test Method** |
| --- | --- | --- | --- | --- |
| PT | 10.7 | S | 9.8-13 | Instrumental Method |
| INR | 0.93 |  | 0.8-1.5 | Calculation Method |
| APTT | 24.2 | S | 24-35 | Instrumental Method |
| Fib | 3.80 | g/L | 2-4 | Instrumental Method |
| TT | 17.5 | S | 12-21 | Instrumental Method |

Table S7. The results of rheumatoid arthritis test panel

| **Inspection items** | **Detection results** | **Units** | **Reference ranges** | **Test Method** |
| --- | --- | --- | --- | --- |
| HS-CRP | 0.90 | mg/L | 0-5 | Immunoturbidimetry |
| RF | 6.60 | IU/mL | 0-18 | Immunoturbidimetry |
| ASO | 2.40 | IU/mL | 0-160 | Immunoturbidimetry |
| CCP | 5.8 | U/mL | 0-46 | Immunoturbidimetry |

Table S8. The results of ANA 7-item panel

| **Inspection items** | **Detection results** | **Units** | **Reference ranges** | **Test Method** |
| --- | --- | --- | --- | --- |
| Ro52 | ＜2.0 | RU/ml | 0-20 | Chemiluminescence Method |
| SSA | ＜2.0 | RU/ml | 0-20 | Chemiluminescence Method |
| SSB | ＜2.0 | RU/ml | 0-20 | Chemiluminescence Method |
| Sm | ＜2.0 | RU/ml | 0-20 | Chemiluminescence Method |
| nRNP/Sm | ＜2.0 | RU/ml | 0-20 | Chemiluminescence Method |
| Scl-70 | ＜2.0 | RU/ml | 0-20 | Chemiluminescence Method |
| Jo-1 | ＜2.0 | RU/ml | 0-20 | Chemiluminescence Method |

Table S9. The results of tumor marker panel(3 tests)+CA-125+CA-153+CA-199+CA72-4

| **Inspection items** | **Detection results** | **Units** | **Reference ranges** | **Test Method** |
| --- | --- | --- | --- | --- |
| AFP | 1.82 | ug/L | ＜7 | Chemiluminescence Method |
| CEA | 1.58 | ug/L | ＜4.5 | Chemiluminescence Method |
| FER | 39.16 | ug/L | 12-135 | Chemiluminescence Method |
| CA-125 | 18.41 | kU/L | ＜35 | Chemiluminescence Method |
| CA-153 | 5.60 | kU/L | ＜15 | Chemiluminescence Method |
| CA-199 | 23.52 | kU/L | ＜30 | Chemiluminescence Method |
| CA72-4 | 0.64 | U/ml | 0-7 | Chemiluminescence Method |

Table S10. The full name of inspection items

| **Abbreviation** | **Full name** |
| --- | --- |
| WBC | White Blood Cell Count |
| NEU# | Absolute Neutrophil Count |
| LY# | Absolute Lymphocyte Count |
| MO# | Absolute Monocyte Count |
| EOS# | Absolute Eosinophil Count |
| BAS# | Absolute Basophil Count |
| NEU% | Neutrophil Percentage |
| LYM% | Lymphocyte Percentage |
| MO% | Monocyte Percentage |
| EOS% | Eosinophil Percentage |
| BAS% | Basophil Percentage |
| RBC | Red Blood Cell Count |
| HBG | Hemoglobin |
| HCT | Hematocrit |
| MCV | Mean Corpuscular Volume |
| MCH | Mean Corpuscular Hemoglobin |
| MCHC | Mean Corpuscular Hemoglobin Concentration |
| PLT | Platelet Count |
| MPV | Mean Platelet Volume |
| PDW | Platelet Distribution Width |
| PCT | Plateletcrit |
| RDW-SD | Red Cell Distribution Width - Standard Deviation |
| RDW-CV | Red Cell Distribution Width - Coefficient of Variation |
| ALT | Alanine Aminotransferase |
| AST | Aspartate Aminotransferase |
| ALP | Alkaline Phosphatase |
| GGT | Gamma-Glutamyl Transferase |
| LDH | Lactate Dehydrogenase |
| TBA | Total Bile Acid |
| GLU | Glucose |
| BUN | Blood Urea Nitrogen |
| CREA | Creatinine |
| UA | Uric Acid |
| K⁺ | Potassium |
| Na⁺ | Sodium |
| Cl⁻ | Chloride |
| FT3 | Free Triiodothyronine |
| FT4 | Free Thyroxine |
| TSH | Thyroid-Stimulating Hormone |
| PT | Prothrombin Time |
| INR | International Normalized Ratio |
| APTT | Activated Partial Thromboplastin Time |
| Fib | Fibrinogen |
| TT | Thrombin Time |
| HS-CRP | High-Sensitivity C-Reactive Protein |
| RF | Rheumatoid Factor |
| ASO | Anti-Streptolysin O |
| CCP | Anti-Cyclic Citrullinated Peptide |
| Ro52 | Ro52 Antibody / Anti-SS-A 52 |
| SSA | Anti-Sjögren's Syndrome A Antibody (Ro60) |
| SSB | Anti-Sjögren's Syndrome B Antibody (La) |
| Sm | Anti-Smith Antibody |
| nRNP/Sm | Anti-nRNP/Sm Antibody (Anti-U1 RNP/Sm) |
| Scl-70 | Anti-Scl-70 Antibody (Anti-Topoisomerase I) |
| Jo-1 | Anti-Jo-1 Antibody (Anti-Histidyl-tRNA Synthetase) |
| AFP | Alpha-Fetoprotein |
| CEA | Carcinoembryonic Antigen |
| FER | Ferritin |
| CA-125 | Cancer Antigen 125 |
| CA-153 | Cancer Antigen 15-3 |
| CA-199 | Cancer Antigen 19-9 |
| CA72-4 | Cancer Antigen 72-4 |
